# Dr. Edward Pollock Anshutz

**Published:** 1918-04

**Authors:** 


### OBITUARY

---

**Readers are requested to report promptly the death of all physicians in Western New York, or former residents of this region, or graduates of any medical school in Western New York, and to notify the families of the deceased of our desire to publish adequate Obituary notices.**

---

**Dr. Edward Pollock Anshutz,** Hering Medical College (Hon.) 1909, died in Philadelphia, Jan. 31, age 71. He was the editor of the Homeopathic Recorder. In December Boericke & Tafel published his book, "New, Old and Forgotten Remedies."

